# Small Fiber Neuropathy in Burning Mouth Syndrome: A Systematic Review

**DOI:** 10.3390/ijms252111442

**Published:** 2024-10-24

**Authors:** Maria Kouri, Daniela Adamo, Emmanouil Vardas, Maria Georgaki, Federica Canfora, Michele Davide Mignogna, Nikolaos Nikitakis

**Affiliations:** 1 Department of Oral Medicine & Pathology and Hospital Dentistry, School of Dentistry, National and Kapodistrian University of Athens, 15772 Athens, Greece; kourimari@dent.uoa.gr (M.K.); emvard@dent.uoa.gr (E.V.); mar1georgaki@gmail.com (M.G.); nnikitakis@dent.uoa.gr (N.N.); 2 Department of Neurosciences, Reproductive and Odontostomatological Sciences, Federico II University of Naples, 80131 Naples, Italy; federica.canfora@unina.it (F.C.); mignogna@unina.it (M.D.M.)

**Keywords:** burning mouth syndrome, small fiber neuropathy, TRPV1, P2X3, Nav1.7, Nav1.9

## Abstract

Burning mouth syndrome (BMS) is a chronic idiopathic orofacial pain disorder, characterized by persistent burning sensations and pain without clear pathological causes. Recent research suggests that small fiber neuropathy (SFN) may play a significant role in the neuropathic pain and sensory disturbances associated with BMS. Following PRISMA guidelines, this systematic review aims to evaluate and synthesize current evidence supporting SFN’s involvement in BMS. The protocol is registered in PROSPERO (CRD42024555839). The results show eight studies reported reductions in nerve fiber density in tongue biopsies (ranging from 30% to 60%), along with morphological changes indicative of small fiber damage. Additionally, an increase in TRPV1-positive, NGF-positive, and P2X3-positive fibers, overexpression of Nav1.7, and slight underexpression of Nav1.9 mRNA were observed in BMS patients. Quantitative Sensory Testing in seven studies revealed sensory abnormalities such as reduced cool detection and cold pain thresholds. Blink reflex and corneal confocal microscopy also indicated peripheral and central small fiber damage, along with increased artemin mRNA expression. The evidence strongly supports SFN as a key factor in the pathophysiology of BMS, particularly due to reductions in nerve fiber density and altered sensory thresholds. However, variability across studies highlights the need for larger, standardized research to establish causal relationships and guide therapeutic strategies.

## 1. Introduction

Burning mouth syndrome (BMS) is an idiopathic, chronic orofacial pain disorder affecting the oral cavity, characterized by a persistent burning sensation and pain, lasting for more than three months, without any local or systemic pathological changes [1]. This condition significantly impacts patients’ daily lives, often leading to difficulties in eating, speaking, and sleeping, which can severely diminish their quality of life [2]. 

Historically, BMS has been referred to by various terms, such as stomatodynia, stomatopyrosis, glossodynia, and oral dysesthesia [3]. The condition typically presents bilaterally, aligning with the trigeminal nerve distribution. Patients with BMS often report a variety of symptoms, including burning, itching, tingling, intraoral foreign body sensation, xerostomia, dysgeusia, globus pharyngeus, and subjective changes in tongue morphology [4]. These symptoms often lead to referrals to multiple medical specialties [4,5] and are commonly associated with mood disorders, sleep disturbances, and cognitive impairments [6,7], further exacerbating the negative impact on patients’ quality of life [2,8,9].

The prevalence of BMS varies significantly, ranging from 1.73% in the general population to 7.72% in clinical settings; in Europe, the prevalence is estimated to be between 5.58% and 6.46% [10]. The disorder is notably more common among postmenopausal women [4,5,6], with a female-to-male ratio of 3:1 [10]. This variability in prevalence may be due to the lack of standardized diagnostic criteria and the unclear neurophysiological mechanisms underlying BMS symptoms [11].

BMS is recognized as a multifactorial neuropathic disease, and its precise etiology remains elusive. However, emerging evidence indicates a dual involvement of central and peripheral pathways; specifically, a strong association with small fiber neuropathy (SFN) [12,13,14,15,16,17].

SFN primarily affects small-diameter sensory fibers, specifically the thinly myelinated Aδ fibers and unmyelinated C fibers [18], leading to chronic neuropathic pain and autonomic dysfunction [19,20,21]. These fibers are essential for pain and temperature perception [22,23]. Aδ fibers, which are myelinated and transmit signals rapidly, respond to non-inflammatory painful stimuli and cold temperatures [24,25]. In contrast, the slower, unmyelinated C fibers react to inflammatory substances and intense stimuli that cause tissue damage [24]. Together, these fibers play a critical role in nociception, thermoreception, and the regulation of autonomic functions [22].

SFN prevalence ranges from 53 per 100,000 people to 2.4%, with higher rates in older adults [26,27]. Common causes of SFN include metabolic diseases like diabetes, genetic disorders, autoimmune diseases, and chronic infections, though many cases remain idiopathic [18,28,29,30]. SFN often presents diagnostic and therapeutic challenges due to its varied etiology and the difficulty in detecting small fiber function through standard electrophysiological tests. This damage leads to neuropathic and burning pain, allodynia, paresthesia, tingling, and loss of pinprick and thermal sensation, as well as various autonomic symptoms [19,20,21] like abnormal sweating, episodic flushing, or gastroparesis [18].

In primary SFN, distinguishing genetic forms from idiopathic forms is essential. Approximately half of SFN cases are idiopathic, with no identifiable cause [31,32,33,34]. Mutations in voltage-gated sodium channel genes (SCN9A, SCN10A, SCN11A, and SCN2B) have been linked to inherited SFN [35,36,37], while autoimmune, inflammatory, infectious diseases, alcohol toxicity, drugs, and metabolic disorders are common causes of acquired SFN [18,30,33]. SFN assessment methods include quantitative sensory tests (QST) and biopsies, which measure the function of Aδ and C fibers [38,39].

Recent research and advancements in diagnostic methodologies have indicated that SFN might play a significant role in BMS, contributing to its neuropathic pain characteristics. Techniques such as skin biopsies for intraepithelial nerve fiber density measurement, along with neurophysiologic methods, have proven highly effective in detecting subclinical abnormalities in patients with BMS. These techniques show a marked reduction in intraepithelial nerve fibers, implicating SFN in the condition’s pathophysiology [16,40].

This systematic review aims to critically examine the literature on the relationship between BMS and SFN. The objective is to synthesize existing evidence, identify knowledge gaps, and provide insights into the pathophysiological links and clinical implications of this association. By rigorously evaluating the available studies, this review seeks to enhance understanding of the interplay between SFN and BMS, thereby improving diagnostic and therapeutic approaches for patients affected by these debilitating conditions.

## 2. Methods

### 2.1. Protocol Registration

This systematic review has been registered in PROSPERO, the International Prospective Register of Systematic Reviews (registration number CRD42024555839). This registration ensures transparency and adherence to predefined methodological standards as outlined by the Center for Reviews and Dissemination at the University of York.

### 2.2. Eligibility Criteria

The research question was developed using the PICOS framework:

- Population (P): Patients diagnosed with burning mouth syndrome (BMS).
- Intervention (I): Diagnostic tests indicative of SFN
- Comparator (C): Control groups including healthy patients referred for dental management without BMS or healthy subjects without BMS.
- Outcome (O): Detection of SFN.
- Study Design (S): Clinical studies focusing on the relationship between BMS and SFN.

### 2.3. Literature Search Strategy

A comprehensive and systematic literature search was conducted following the Preferred Reporting Items for Systematic Reviews and Meta-Analyses (PRISMA) guidelines [41]. The search was performed across multiple databases—PubMed, Scopus, and Web of Science—on 18 June 2024, without any restrictions on publication year to ensure an exhaustive review of the available literature. 

To identify relevant studies, the following Medical Subject Headings (MeSH) and keywords were used: “burning mouth syndrome”, “burning tongue”, “oral burning”, “glossalgia”, “glossodynia”, “glossopyrosis”, “stomatodynia”, “stomatopyrosis”, “sore mouth”, “sore tongue”, “oral dysesthesia”, “BMS”, and “small fiber neuropathy”. For the Web of Science database, additional filters were applied, including “MeSH terms” and “search within results”, to refine the search outcomes.

Additionally, manual searches of the reference lists from the retrieved articles were conducted to identify any other potentially eligible studies that might have been missed in the database searches.

### 2.4. Inclusion and Exclusion Criteria

Studies were included if they met the following criteria: (1) focused on the clinical association between BMS and SFN, (2) were published in peer-reviewed journals, (3) included control groups for comparison, and (4) were written in English. Studies were excluded if they were reviews, case reports, or conference papers, or did not specifically assess SFN in BMS patients. We also performed a rigorous screening of studies to ensure relevance to the research question, and objective studies were excluded for the following reasons: (1) reviews, case reports, conference papers, letters to the editor, expert opinions, or comments; (2) studies published in languages other than English; (3) studies lacking control or comparison groups; and (4) studies that did not specifically assess SFN in patients with BMS. In addition, studies were excluded if they failed to meet the inclusion criteria after full-text evaluation, ensuring objectivity and relevance. 

To minimize bias in the screening process, multiple reviewers independently evaluated the abstracts and full texts, with any discrepancies resolved through consensus discussions. A detailed flow diagram (see Supplementary Materials) was provided to clearly show the selection and exclusion process, adding transparency to the methodology.

### 2.5. Study Selection Process

To ensure rigor in the selection process, duplicate studies were first removed. Subsequently, the titles and abstracts of the remaining articles were independently screened for eligibility by two reviewers (MK, DA). Any discrepancies in their decisions were resolved through consultation with a third reviewer (NN). For the articles deemed potentially relevant, full-text reviews were conducted independently by two reviewers (MK, EP), who were blinded to each other’s decisions. Disagreements during this stage were adjudicated by a third reviewer (MM) using a structured decision process documented in Microsoft Excel 2023 (Redmond, WA, USA).

A detailed flow diagram, along with a comprehensive summary of excluded studies and the reasons for their exclusion, is provided in the Supplementary Document (see Supplementary Materials). This information serves to clearly illustrate the selection and exclusion process, ensuring transparency and rigor in our methodology. 

### 2.6. Data Extraction 

Following the identification of eligible studies, relevant data were meticulously extracted from each article. The extracted data were categorized as follows:

Study-related data: Including the first author, study design, sample size, and participants’ age in both BMS and control groups.

Study characteristics: Detailing the type of diagnostic test used, site of application, and specific methodological approaches.

Outcome-related data: Focusing on relevant outcomes and the implications for SFN.

A designated individual oversaw the data management process, using a structured data extraction table created in Microsoft Excel to systematically record and organize information across a comprehensive range of domains.

Due to significant heterogeneity among the included studies, a meta-analysis was not conducted, and data synthesis was based on the type of test used to assess SFN involvement.

### 2.7. Quality Assessment 

The quality of the included studies was assessed using the NIH Quality Assessment Tool for case-control studies [42]. The overall risk of bias ranged from 25% to 67%, with common issues such as lack of sample size justification, inadequate blinding, and unclear control group selection. This indicates that while the studies provide valuable insights, the moderate level of bias necessitates cautious interpretation of the results, particularly for studies like Grushka et al. [43] and Domaneschi et al. [44], which showed higher risks of bias.

Two reviewers (MK, EV), blinded to each other’s assessments, independently performed the evaluation. Disagreements in quality ratings were resolved by a third reviewer (NN), ensuring consistency and objectivity. 

## 3. Results and Discussion

**Figure 1:**
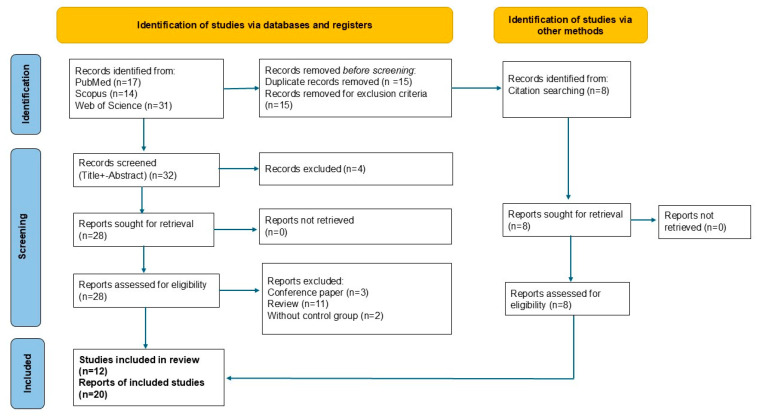
Prisma (2021) flow diagram of the systematic review process. The diagram shows the number of records identified from various databases (PubMed, Scopus, Web of Science) and citation searches, and illustrates how studies were screened, assessed for eligibility, and excluded based on predefined criteria. A total of 32 records were screened, leading to 12 studies being included in the final review, along with 20 reports of included studies. Exclusion reasons include conference papers, reviews, and studies without control groups.

The initial search process identified 62 records from the following databases: PubMed, Scopus, and Web of Science. After removing 15 duplicates and 15 records considering the exclusion criteria, 32 unique records remained for screening. These records were screened based on their titles and abstracts, resulting in the exclusion of four articles that did not meet the inclusion criteria. This left 28 full-text articles for detailed assessment. During the full-text review, 16 articles were excluded for reasons such as being review articles, lacking a control group, or not focusing on SFN in BMS. Additionally, eight relevant articles were identified through a manual search of reference lists from the retrieved articles. After the full screening and review process, a total of 20 studies met the criteria and were included in the qualitative synthesis (Table 1). These studies were rigorously selected to ensure their relevance and methodological robustness, as depicted in the PRISMA flow diagram (Figure 1).

Table 2 presents a comprehensive summary of the participant characteristics and the diagnostic tests utilized across the 20 included studies [16,17,40,43,44,45,46,47,48,49,50,51,52,53,54,55,56,57,58,59], all of which were prospective with control groups. Across all studies, a total of 409 BMS patients were included while the control groups comprised a total of 309 participants, matched appropriately in size to the BMS cohorts. The studies varied in sample size, with the number of BMS participants ranging from 5 to 45, and control groups were appropriately matched in size to the BMS cohorts, ensuring reliable comparisons. The mean age of participants typically fell within the middle-aged to elderly range, with most studies reporting mean ages between 50 and 70 years, which aligns with the demographic most affected by BMS [10].

The studies employed a variety of diagnostic techniques to assess different aspects of SFN involvement in BMS. QST was used in seven studies [43,45,46,51,54,55,56,57] to measure sensory thresholds related to small fiber function. Biopsies were conducted in another seven studies [16,17,46,47,48,50,53] to evaluate nerve fiber density and structural changes in the tissues affected by BMS. Additionally, blink reflex (BR) assessments were utilized in one study [52] to assess the integrity of nerve pathways and reflexive responses, while corneal confocal microscopy (CCM) was applied in one study [58] to examine the corneal nerve fibers, reflecting potential systemic small fiber damage. Capsaicin testing was used in one study [49] to assess peripheral nerve sensitivity by applying capsaicin to the tongue and measuring the response, and Artemin mRNA expression analysis was used in one study [55] to explore molecular changes in the expression of artemin, a factor involved in nerve growth and function. Furthermore, two studies employed multiple techniques: one combined QST, BR, and biopsy [40] and another used both QST and BR [59] to provide a more comprehensive assessment.

Table 3 highlights the findings from studies that utilized QST to assess SFN in patients with BMS. Most of the studies (7 out of 9) [43,45,46,54,56,57,59] provided evidence supporting the involvement of SFN in BMS, characterized by sensory abnormalities such as increased thermal thresholds, decreased heat pain tolerance, and signs of peripheral nerve fiber degeneration. 

Grushka et al. [43] and Svensson et al. [45] reported significantly lower heat pain tolerance in BMS patients compared to controls, despite similar thermal detection thresholds, suggesting altered pain processing, potentially linked to Aδ fiber dysfunction.

Similarly, Ito et al. [46] observed elevated thermal pain thresholds in various regions of the tongue, further indicating peripheral nerve dysfunction.

Mo et al. [54] provided evidence for SFN involvement by showing significantly lower cold detection thresholds (CDT) and cold pain thresholds (CPT), along with higher heat pain thresholds (HPT), consistent with thermal sensory loss typical of SFN.

Yilmaz et al. [56] also found significantly lower CDT, warm detection thresholds (WDT), and CPT, suggesting ion channel impairments in Aδ and C fiber nerve endings.

Hartmann et al. [57] and Kolkka et al. [59] reported sensory abnormalities, with Hartmann et al. [57] identifying higher CDT and WDT, and lower CPT, while Kolkka et al. [59] found elevated WDT and CDT.

In contrast, Kaplan et al. [51] found no significant differences in WDT, CDT, HPT, or CPT between BMS patients and controls. Puhakka et al. [40] also reported mixed results, with significantly higher CDT but no corresponding changes in WDT or HPT.

Table 4 summarizes the findings from 8 studies [16,17,40,46,47,48,50,53] that utilized biopsies to assess SFN in patients with BMS. Six out of eight biopsy studies [16,17,40,47,50,56] reported significant reductions in intraepithelial nerve fiber density in patients with BMS, providing strong evidence for the involvement of SFN in its pathophysiology. 

Lauria et al. [16] observed a 40% decrease in nerve fiber density in BMS patients, alongside morphological changes suggestive of trigeminal SFN. Similarly, Yilmaz et al. [56] found a notable reduction in intraepithelial nerve fibers, combined with increased levels of TRPV1-positive and nerve growth factor (NGF) fibers, which correlated with pain severity. Beneng et al. [47] identified increased P2X3-positive fibers and reduced neurofilament-staining fibers in the tongue mucosa of BMS patients, further supporting SFN involvement. 

However, in a subsequent study, Beneng et al. [48] observed only a trend toward an increase in Nav1.7 immunoreactive fibers in BMS, but this was not statistically significant, suggesting some ambiguity in the role of these voltage-gated sodium channels.

Penza et al. [50] and Borsani et al. [53] also documented significant reductions in nerve fiber density, while Puhakka et al. [40] confirmed lower intraepithelial nerve fiber density compared to controls, consistent with small fiber damage.

Domaneschi et al. [46] found no significant differences in the expression of Nav1.7 or Nav1.9 channels in BMS patients, although their data suggested potential dysregulation of these sodium channels.

Table 5 summarizes the findings from three studies that utilized BR assessments to SFN in patients with BMS. All three studies [40,52,59] consistently revealed evidence of SFN, either as a focal issue or as part of a broader neuropathic process involving both peripheral and central mechanisms. 

Mendak et al. [52] reported significant differences in BR parameters, including prolonged latencies and frequent irregularities, which indicate mild sensory and autonomic small fiber neuropathy with potential central nervous system involvement. 

Puhakka et al. [40] observed longer BR latencies in BMS patients compared to healthy controls, although the changes were not statistically significant compared to healthy controls, suggesting the presence of peripheral small fiber damage that may not be isolated to the small fiber system alone. Similarly, Kolkka et al. [59] found increased stimulation thresholds and decreased neurophysiological function of Aδ fibers in BMS patients, further supporting the involvement of SFN in BMS. However, the results were not always statistically significant, highlighting some variability in BR responses.

Table 6 presents findings from three studies that employed unique methodologies to assess SFN in patients with BMS. Just et al. [49] conducted a study using capsaicin-impregnated filter-paper strips applied to the dorsal anterior tongue, which revealed that BMS patients exhibited higher pain thresholds and elevated sensation-related thresholds, indicating impaired small fiber function. Shinoda et al. [55] investigated artemin mRNA expression in the tongue mucosa epithelial cells and found a significant increase in BMS patients, suggesting enhanced activity of heat-sensitive nerve fibers. Additionally, O’Neill et al. [58] used CCM to detect corneal small fiber damage in BMS patients. Their findings showed reduced corneal nerve fiber density and length, as well as an increased number of Langerhans cells, further indicating corneal small fiber damage.

The results of this systematic review provide compelling evidence supporting the involvement of SFN in the pathogenesis of BMS. The comprehensive analysis of 20 clinical studies [16,17,40,43,44,45,46,47,48,49,50,51,52,53,54,55,56,57,58,59], including a total of 409 BMS patients and 309 controls has revealed consistent patterns of sensory dysfunction, nerve fiber density reduction, and morphological changes in BMS patients that are indicative of SFN. Diagnostic methods such as QST, biopsies, and BR assessments were commonly employed. The participant demographic predominantly fell within the middle-aged to elderly range (50 to 70 years), aligning with the population most affected by BMS [10] and enhancing the generalizability of the findings. 

The moderate risk of bias in many studies suggests that the findings, though indicative, should be interpreted with caution. Common issues, such as inadequate sample size justification, inadequate blinding, and unclear control group selection, were especially notable in studies with higher bias scores. While most studies clearly stated their objectives, defined populations, and included appropriate controls, key limitations persisted. These included a lack of random selection or blinded assessors, and in some cases, vague population definitions, as observed in Grushka et al. [43]. Although many studies recruited control groups from comparable populations—thus minimizing confounding variables—some, like Ito et al. [46], did not clearly report control group selection, raising concerns about selection bias. Despite these shortcomings, the majority of studies met basic inclusion criteria and were rated as “fair” quality.

### 3.1. QST and Sensory Dysfunction in BMS

The results from the majority of QST studies [43,45,46,54,56,57,59] strongly indicate that SFN plays a significant role in BMS, as evidenced by altered thermal thresholds, reduced heat pain tolerance, and signs of peripheral nerve fiber degeneration. The observation of both increased and decreased CDT and WDT suggests that BMS patients may experience either hypersensitivity or hyposensitivity to thermal stimuli, implicating dysfunction in both Aδ and C fibers. This is consistent with previous findings, such as the meta-analysis by Madariaga et al. [60], which reported thermal threshold alterations in 38.5% of the studies reviewed.

The pattern of sensory abnormalities observed in BMS aligns with findings in other SFN-related conditions, such as diabetic neuropathy, fibromyalgia, and idiopathic small fiber neuropathy (ISFN) [61], where patients often present with increased thermal detection thresholds and reduced pain sensitivity.

However, the variability in QST results suggests that SFN may not be present in all BMS patients. Kaplan et al. [51], for example, found no significant differences in CDT or WDT between patients and controls, indicating that SFN might not be the only underlying mechanism in BMS.

Additionally, the mixed outcomes reported by Puhakka et al. [40], where only CDT was elevated, suggest that SFN in BMS may not consistently affect all sensory modalities. This variability might also point to the involvement of central neuropathic mechanisms, such as conditions like fibromyalgia, where central sensitization plays a key role [16,62]. Therefore, BMS should be regarded as a heterogeneous condition, with SFN being a central component in some patients but not in others.

### 3.2. Nerve Fiber Density and Morphological Changes

The results from six of eight biopsy studies [16,17,40,47,50,56] strongly suggest that SFN plays a critical role in BMS, as evidenced by the significant reduction in intraepithelial nerve fiber density. This reduction parallels findings in other SFN-related conditions, such as diabetic neuropathy and ISFN [63,64], which are characterized by similar sensory disturbances. The loss of small nerve fibers in BMS likely contributes to the burning pain and sensory abnormalities reported by patients, indicating disrupted sensory signaling and altered pain and temperature perception. 

Further supporting these findings, Yilmaz et al. [56] and Beneng et al. [47] identified increased expression of TRPV1 and P2X3 receptors, nociceptive markers commonly associated with pain and temperature regulation. TRPV1, known for its role in detecting and regulating body temperature and pain, is often upregulated in chronic pain conditions. 

Although nerve fiber loss is characteristic of SFN, the upregulation of P2X3 receptors likely occurs in the remaining or regenerating fibers. This phenomenon is consistent with compensatory mechanisms observed in other neuropathic conditions, where surviving neurons increase receptor expression in response to nerve damage. This upregulation may enhance the sensitivity to pain stimuli despite the overall reduction in small sensory neurons, thereby contributing to the persistent burning sensations experienced by BMS patients [65].

While Domaneschi et al. [46] did not find statistically significant differences in the expression of Nav1.7 or Nav1.9 sodium channels in BMS patients, other studies suggest a potential involvement of these channels in the pathogenesis of neuropathic pain. Nav1.7, in particular, has been implicated in various neuropathic pain disorders, such as erythromelalgia and SFN [66,67], where overexpression can heighten pain perception. Therefore, even in the absence of significant expression changes, the role of these channels in abnormal pain signaling should not be ruled out. Further studies are needed to explore the precise contribution of sodium channels in BMS pathophysiology.

Overall, these findings align with broader SFN literature, where nerve fiber loss and receptor upregulation are common features. The patterns observed in BMS—nociceptor upregulation and small fiber loss—mirror those seen in conditions such as fibromyalgia and diabetic neuropathy [62,63], suggesting a shared mechanism underlying chronic pain and sensory disturbances.

### 3.3. Blink Reflex and Other Diagnostic Techniques

The BR assessments provided valuable insights into the pathology of BMS, revealing evidence of SFN in most studies [40,52,59], with prolonged latencies and irregularities suggesting peripheral small fiber damage and potential central nervous system involvement. 

Mendak et al. [52] reported significant differences in BR parameters, such as prolonged latencies and frequent irregularities, indicative of mild sensory and autonomic small fiber neuropathy with potential central nervous system involvement. These findings align with other SFN-related conditions, including trigeminal neuralgia and idiopathic facial pain, where similar BR abnormalities suggest the involvement of small-diameter fibers [68].

However, the variability in BR findings, as seen in studies by Puhakka et al. [40] and Kolkka et al. [59], suggests that SFN in BMS may not uniformly affect all sensory modalities. This inconsistency in results, also observed in ISFN [69], reflects the complexity and heterogeneity of SFN presentation in BMS. The lack of significant BR differences in some cases may indicate that BR testing, while useful, lacks the sensitivity to detect all forms of SFN, or that the condition affects different nerve fibers at different stages.

Additionally, Kolkka et al. [59] identified negative neurophysiological signs indicative of decreased Aδ fiber function, further supporting the presence of peripheral small fiber damage and a neuropathic pain condition due to focal SFN. Notably, the study found that benzodiazepines or other central nervous system-affecting drugs did not influence stimulation thresholds in BMS patients, suggesting that the neuropathic pain and SFN in BMS may be more localized to peripheral pathways rather than being significantly modulated by central interventions.

The absence of significant BR abnormalities in most BMS patients compared to controls implies that BMS may involve more complex or variable pathophysiological mechanisms than can be detected through BR testing alone. This finding is consistent with broader SFN literature [70,71], where diagnostic tools like BR often yield variable results depending on the specific fibers affected and the extent of nerve involvement. For instance, in conditions like ISFN and trigeminal neuralgia [72], BR abnormalities are not universally observed, suggesting that the utility of BR may be limited to specific subtypes of SFN or stages of disease progression. These findings support the hypothesis that BMS involves focal SFN, like patterns observed in conditions such as diabetic neuropathy [63], where BR testing has revealed dysfunctions related to small fiber damage. The variability in BR responses across different studies and stimulation sites underscores the complex and multifactorial nature of SFN in BMS, suggesting that a combination of peripheral and central mechanisms may be involved.

In conclusion, while BR assessments contribute valuable insights into the involvement of SFN in BMS, their diagnostic utility may be limited by variability in sensitivity and specificity across different patient populations and stages of disease. BR testing should therefore be used as a complementary tool within a broader diagnostic framework, considering the diverse clinical presentations and underlying mechanisms of SFN in BMS.

### 3.4. Other Diagnostic Techniques

Recent advances in diagnostic methodologies, such as capsaicin-impregnated strips, artemin mRNA expression analysis, and CCM, have reinforced the potential link between BMS and SFN. These techniques offer a multifaceted approach to understanding the underlying neuropathic processes, emphasizing the role of small fiber dysfunction in the altered sensory perceptions experienced by BMS patients.

The study by Just et al. [49] found higher pain thresholds and reduced sensation-related thresholds in BMS patients, suggesting impaired small fiber function in the peripheral trigeminal system. Capsaicin, which activates TRPV1 receptors on small sensory fibers, particularly C-fibers and Aδ-fibers [73], revealed desensitization or dysfunction of these fibers, leading to altered pain perception. This supports the hypothesis that BMS is associated with peripheral trigeminal sensitivity issues, implicating SFN as a contributing factor in the altered sensory experiences of BMS patients.

Shinoda et al. [55] demonstrated increased artemin mRNA expression in the tongues of BMS patients, suggesting a molecular mechanism contributing to the hypersensitivity and pain observed in BMS. Artemin, a member of the glial cell line-derived neurotrophic factor (GDNF) family, supports the survival and function of small-diameter sensory neurons, particularly C-fibers, by binding to the GDNF family receptor α3 (GFRα3) [74,75]. The upregulation of artemin could enhance GFRα3 signaling, leading to the recruitment and sensitization of heat-sensitive fibers, contributing to heightened pain sensitivity indicating an underlying neuropathic process affecting small fibers. These findings align with research by Elitt et al., who demonstrated that in transgenic mice overexpressing artemin in skin keratinocytes, there was a corresponding increase in TRPV1 expression in dorsal root ganglion neurons, linking artemin-GFRα3 signaling to increased nociceptive responses [76]. Furthermore, Shinoda et al.’s study found that a mouse model of BMS exhibited increased artemin protein expression in the keratinized epithelium of the tongue, indicating a direct role of artemin in the neuropathic pain mechanisms associated with BMS. Notably, when anti-artemin neutralizing antibodies were administered in the tongues of these mice, there was a reversal of heat hyperalgesia and a reduction in the number of TRPV1-positive and GFRα3-positive trigeminal neurons. These results suggest that artemin may serve as a potential biomarker for SFN in BMS, reflecting its pivotal role in the pathogenesis of this condition [55].

O’Neill et al. [58] utilized CCM to examine the corneal sub-basal nerve plexus in BMS patients, revealing significantly lower corneal nerve fiber density and length, along with an increased number of Langerhans cells. The reduction in nerve fiber density and length indicates small fiber damage, while the rise in Langerhans cells, which are antigen-presenting cells [77], may reflect an inflammatory process contributing to the neuropathy. These findings reinforce the hypothesis that BMS involves small fiber damage not just in the oral cavity but also in other regions like the cornea, suggesting a more systemic involvement of small fibers.

These findings align with other studies on SFN-related pathologies. For instance, Quattrini et al. [78] found similar reductions in nerve fiber density in diabetic neuropathy using CCM, with corneal nerve fiber length and density serving as predictors of disease severity and progression in chronic idiopathic axonal neuropathy [70]. Additionally, hereditary sensory and autonomic neuropathies have been diagnosed early using CCM and capsaicin testing, enabling earlier intervention [70]. Increased artemin levels have also been linked to heightened pain sensitivity and inflammation in conditions like rheumatoid arthritis [71].

The use of different diagnostic modalities—such as capsaicin testing, artemin mRNA expression, and corneal confocal microscopy—provides complementary evidence supporting this hypothesis of SFN in BMS. These techniques highlight different aspects of small fiber dysfunction, from altered pain thresholds to molecular changes and structural damage, offering a more comprehensive understanding of the neuropathic components of BMS. Furthermore, the utility of these methods in other neuropathic conditions underscores their broader relevance and potential for improving diagnostic accuracy and patient outcomes across various SFN-related pathologies.

The combination of capsaicin testing, artemin mRNA expression, and CCM provides complementary evidence supporting SFN as a key factor in BMS. These techniques highlight various aspects of small fiber dysfunction, ranging from altered pain thresholds to molecular changes and structural damage, offering a more comprehensive understanding of the neuropathic components of BMS. Furthermore, their successful use in other neuropathic conditions underscores their broader relevance and potential to enhance diagnostic accuracy and treatment outcomes for SFN-related pathologies.

The management of SFN in BMS remains a significant challenge in oral medicine, as it involves addressing both neuropathic pain and underlying nerve dysfunction. Pharmacological interventions, such as anticonvulsants (e.g., gabapentin, pregabalin), tricyclic antidepressants, and topical agents like capsaicin, are frequently employed due to their demonstrated efficacy in alleviating neuropathic pain, particularly in the context of peripheral neuropathy [79]. These treatments may offer comparable benefits in BMS by modulating nerve hyperexcitability and reducing the pain signals typically associated with SFN.

Topical treatments, such as clonazepam, have gained attention for their ability to desensitize TRPV1 receptors, which are implicated in the burning sensations experienced by BMS patients. Studies suggest that individuals with BMS exhibit elevated TRPV1 expression, making TRPV1 receptor modulation a promising therapeutic target [17]. In addition to existing treatments, emerging pharmacotherapies like sodium channel blockers are being explored due to their capacity to alleviate pain by targeting the hyperexcitability of damaged peripheral nerves, as demonstrated in various neuropathic conditions [80].

However, considering the heterogeneity of BMS presentations and underlying mechanisms, individualized treatment plans that integrate both pharmacological and non-pharmacological strategies are essential for optimizing patient outcomes. This is particularly relevant when managing peripheral neuropathy within BMS, as personalized care models allow for more targeted interventions, improving the efficacy of treatment across different patient profiles. In the context of a systematic review, understanding the breadth of pharmacological and non-pharmacological treatments, alongside their effectiveness in treating SFN-related neuropathy within BMS, is crucial for synthesizing current evidence and guiding future research directions.

### 3.5. Limitations

This systematic review has several limitations that should be acknowledged. First, despite the comprehensive nature of the literature search, the review only included studies published in English and indexed in specific databases, potentially missing relevant studies published in other languages or not indexed in these databases. This language and database limitation could introduce selection bias and affect the comprehensiveness of the findings. Second, the studies included in this review involved a relatively small number of BMS patients, with the total sample size across all studies being limited. This small sample size creates a low statistical power for detecting significant differences and limits the generalizability of the findings. Additionally, the heterogeneity in study designs, patient populations, and diagnostic methodologies, particularly in the use of QST protocols, may contribute to the variability in results observed across studies. This heterogeneity also prevented the performance of a meta-analysis, which could have provided a more robust quantitative synthesis of the data. Third, the risk of bias in the included studies ranged from 25% to 67%, indicating that many studies had methodological limitations, such as the lack of sample size justification, random selection, or blinding of assessors. These factors introduce potential biases that could influence the reliability of the findings.

## 4. Conclusions

The collective evidence strongly indicates that SFN plays a significant role in the pathophysiology of BMS, though it is unlikely to be the sole contributor. The variability in findings across studies highlights the heterogeneous nature of BMS, suggesting it may represent a spectrum of conditions with diverse underlying causes, rather than a single, uniform disorder. Factors such as differences in study design, patient populations, diagnostic criteria, and methodologies likely contribute to this variability, complicating our understanding of BMS and indicating the involvement of multiple mechanisms, both peripheral and central.

Notably, robust data from biopsies and molecular analyses have identified peripheral nerve alterations, including the increased expression of nociceptive markers such as TRPV1, P2X3, and Nav1.7, which suggest ongoing peripheral sensitization. These findings align BMS with other neuropathic pain conditions characterized by dysregulated ion channels and receptor overexpression. However, the interplay between peripheral and central mechanisms adds further complexity to the diagnosis and treatment of BMS, highlighting the need for more sophisticated diagnostic approaches.

While evidence supports the role of SFN in BMS, establishing definitive causality requires longitudinal studies. The predominance of cross-sectional research limits our ability to determine temporal relationships between SFN and BMS onset. Future research should aim to address these gaps by employing systematically designed studies with larger, more diverse patient populations, standardized diagnostic criteria, and advanced molecular techniques.

Understanding the role of SFN in BMS opens promising avenues for therapeutic intervention, particularly through treatments targeting small fiber function and neuropathic pathways. By advancing our knowledge of both peripheral and central neural contributions to BMS, we can pave the way for more effective, targeted therapies that may reduce the reliance on broad-spectrum pain management medications and their associated side effects.

Future research should focus on systematically designed studies that include larger, more diverse patient populations, standardized diagnostic criteria, and advanced molecular techniques to confirm SFN’s role and explore additional neuropathic pathways. A deeper understanding of peripheral and/or central neural implications in BMS pathophysiology could lead to more targeted therapeutic approaches, potentially avoiding the side effects associated with commonly prescribed pain management medications.

## Figures and Tables

**Table 1:** Quality assessment of the included studies.

	Grushka et al. 1987 [43]	Svensson et al. 1993 [45]	Ito et al., 2002 [46]	Lauria et al., 2005 [16]	Yilmaz et al., 2007 [17]	Beneng et al., 2010 [47]	Beneng et al., 2010b [48]	Just et al., 2010 [49]	Penza et al., 2010 [50]	Kaplan et al., 2011 [51]	Mendak et al., 2012 [52]	Borsani et al., 2014 [53]	Mo et al., 2015 [54]	Shinoda et al., 2015 [55]	Puhakka et al., 2016 [40]	Yilmaz et al., 2016 [56]	Hartmann et al., 2017 [57]	O’ Neil et al., 2018 [58]	Kolkka et al., 2019 [59]	Domaneschi et al., 2023 [46]
**Criteria**																				
1. Was the research question or objective in this paper clearly stated and appropriate?	y	y	y	y	y	y	y	y	y	y	y	y	y	y	y	y	y	y	y	y
2. Was the study population clearly specified and defined?	n	y	y	y	y	y	y	y	y	y	y	y	y	y	y	y	y	y	y	y
3. Did the authors include a sample size justification?	n	n	n	n	n	n	n	n	n	n	n	n	n	n	n	n	n	y	n	n
4. Were controls selected or recruited from the same or similar population that gave rise to the cases (including the same timeframe)?	nr	y	y	n	y	y	y	y	n	y	y	y	y	y	y	y	y	y	y	y
5. Were the definitions, inclusion and exclusion criteria, algorithms, or processes used to identify or select cases and controls valid, reliable, and implemented consistently across all study participants?	nr	y	y	n	y	y	y	y	cd	y	y	y	y	y	y	y	y	y	y	y
6. Were the cases clearly defined and differentiated from controls?	nr	y	y	y	y	y	y	y	y	y	y	y	y	y	y	y	y	y	y	y
7. If less than 100 percent of eligible cases and/or controls were selected for the study, were the cases and/or controls randomly selected from those eligible?	n	n	n	n	n	n	n	n	n	n	n	n	n	n	n	n	n	n	n	n
8. Was there use of concurrent controls?	y	y	y	y	y	y	y	y	n	y	y	y	y	y	y	y	y	y	y	y
9. Were the investigators able to confirm that the exposure/risk occurred prior to the development of the condition or event that defined a participant as a case?	y	y	y	y	y	y	y	y	y	y	y	y	y	y	y	y	y	y	y	y
10. Were the measures of exposure/risk clearly defined, valid, reliable, and implemented consistently (including the same time period) across all study participants?	y	y	y	y	y	y	y	y	n	y	y	y	y	y	y	y	y	y	y	y
11. Were the assessors of exposure/risk blinded to the case or control status of participants?	n	n	n	n	n	n	n	n	n	n	n	n	n	n	n	n	n	n	n	n
12. Were key potential confounding variables measured and adjusted statistically in the analyses? If matching was used, did the investigators account for matching during the study analysis?	na	na	na	na	na	na	na	na	na	na	na	na	na	na	na	na	na	na	na	na
**Quality**	**f**	**f**	**f**	**f**	**f**	**f**	**f**	**f**	**f**	**f**	**f**	**f**	**f**	**f**	**f**	**f**	**f**	**f**	**f**	**f**
**Risk of bias %**	**67**	**34**	**34**	**50**	**34**	**34**	**34**	**34**	**67**	**34**	**34**	**34**	**34**	**34**	**34**	**34**	**34**	**25**	**34**	**34**

y: yes; n: no; nr: not reported; na: not applicable; cd: cannot determine f: fair. Quality is rated as good (<25% risk of bias), fair (26–74% risk of bias), and poor (>75% risk of bias).

**Table 2:** Characteristics of the 20 full-text studies; number and age of participants, and conducted tests.

Author	Number of Participants (Women)	Mean Age (Range)	Test
Grushka et al. 1987 [43]	BMS: 40 (NA) Control—no oral burning: 23 (NA)	NA	QST
Svensson et al. 1993 [45]	BMS:23 (22)	64 (50–87)	QST
	HC: 23 (21)	68 (46–81)	
Ito et al., 2002 [46]	BMS:20 (20)	52 (43–64)	QST
	HC: 20 (20)	49 (35–59	
Lauria et al., 2005 [16]	BMS: 12 (11) HC: 9 (NA)	NA	Biopsy
Yilmaz et al., 2007 [17]	BMS: 10 (5)	62 (48–82)	Biopsy
	Control—patients attending for wisdom tooth removal: 10 (4)	40 (16–79)	
Beneng et al., 2010 [47]	BMS: 9 (6)	62.4 (NA)/	Biopsy
	Control—patients attending for wisdom tooth removal: 10 (4)	40.8 (NA)	
Beneng et al., 2010 [48]	BMS: 7 (5)	62 (48–82)	Biopsy
	HC: 10 (4)	40 (16–79)	
Just et al., 2010 [49]	BMS: 13 (9)	62 (41–71)	Capsaicin Strips
	HC: 28 (18)	51 (41–63)	
Penza et al., 2010 [50]	BMS: 38 (33)	65.6 (NA)	Biopsy
	Pain in the tip syndrome: 13 (11)	62 (NA)	
	HC: 9 (NA)	NA (NA)	
Kaplan et al., 2011 [51]	BMS: 26 (NA) HC: 43 (NA)	NA	QST
Mendak et al., 2012 [52]	BMS: 33 (27)	61.5 (41–82)	BR
	HC: 30 (22)	60.5 (42–83)	
Borsani et al., 2014 [53]	BMS: 8 (8) HC: 8 (8)	67.5 (54–85)	Biopsy
Mo et al., 2015 [54]	BMS: 25 (17)	49.5 (NA)	QST
	HC: 19 (NA)	47.7 (NA)	
Shinoda et al., 2015 [55]	BMS: 9 (9)	71.6 (58–80)	Artemin mRNA expression
	HC: 9 (5)	75.0 (51–85)	
Puhakka et al., 2016 [40]	BMS: 10 (10)	67.9 (60–77.5)	QST, BR Biopsy
	HC: 10 (10)	67.4 (58.4–75.9)	
	Cadaver control: 13 (13)	64.5 (55.0–72.0)	
	Cadaver control with diabetes: 6 (6)	68.8 (59.0–78.0)	
Yilmaz et al., 2016 [56]	BMS: 22 (14)	57.8 (29–83)	QST
	HC: 17 (11)	46.88 (30–79)	
Hartmann et al., 2017 [57]	BMS: 5 (4)	51.8 (37–70)	QST
	HC: 8 (8)	56.9 (37–69)	
O’ Neil et al., 2018 [58]	BMS: 17 (15)	61.7 (18–85)	CCM
	HC: 14 (7)	59.3 (18–85)	
Kolkka et al., 2019 [59]	BMS: 45 (43)	63.8 (45–82)	QST BR
	HC: 32 (30)	64.8 (48–84)	
Domaneschi et al., 2023 [46]	BMS: 12 (12)	61.4 (48–71)	Biopsy
	Control (healthy borders of benign tongue lesion): 5 (4)	63.4 (48–80)	

Abbreviations: BMS: burning mouth syndrome; BR: blink reflex; HC: healthy control; QST: quantitative sensory testing; CCM: corneal confocal microscopy; NA: not available.

**Table 3:** Studies using QST for SFN assessment.

Author	Test	Site	Relevant Outcomes	SFN Implication Comments
Grushka et al. 1987 [43]	QST: Thermal stimuli between 34–46 °C in 2 °C steps	Tongue tip, lower lip mucosa	No significant difference in the thermal change detection threshold between BMS and controls. Heat pain tolerance significantly lower in BMS than in controls	No significant differences in thermal change detection thresholds between BMS patients and controls Lower heat pain tolerance in BMS patients, suggesting that while Aδ and C fibers may respond similarly to temperature changes, there might be an abnormal pain processing, particularly involving Aδ fibers
Svensson et al. 1993 [45]	QST: Brief argon laser stimulation (2.15 W, 200 ms)	Tongue tip, lower lip mucosa, buccal mucosa, anterior part of hard palate	Significantly increased sensory across various oral and facial sites in BMS patients Significantly decreased heat pain tolerance at tongue tip in BMS patients	Increased sensory thresholds and decrease of heat pain tolerance suggest potential degeneration of peripheral nerve fibers
Ito et al., 2002 [46]	QST: Thermal stimuli between 0–50 °C; Mechanical stimulation	Tongue	Higher thermal pain thresholds (apex and left and right margins of the tongue) in BMS patients compared to controls	Higher thermal pain thresholds may indicate peripheral neurophysiological dysfunction
Kaplan et al., 2011 [51]	QST: Thermal stimuli between 8–50 °C	Middle anterior dorsal tongue surface	No differences in WDT and CDT, HPT and CPT between BMS and healthy controls	The lack of difference in WDT, CDT, HPT, and CPT suggests that SFN may not be a defining feature of BMS highlighting the potential variability in the condition
Mo et al., 2015 [54]	QST: Thermal stimuli between 0–50 °C	Tip of the tongue; lower lip mucosa	Significantly lower CDT and CPT in BMS Significantly higher HPT in BMS	Localized loss of thermal function supports the hypothesis that BMS could be a neuropathic pain condition with the involvement of peripheral and/or central pain mechanisms
Puhakka et al., 2016 [40]	QST: Thermal stimuli between 10–50 °C	Lingual nerve distribution, bilateral	Significantly higher CDT in BMS No significant changes in WDT and HPT	Peripheral neuropathy in BMS might not be confined to small fiber systems alone, potentially involving other nerve fibers or central mechanisms as well
Yilmaz et al., 2016 [56]	QST: Thermal stimuli between 0–50 °C	Anterior two-thirds of the tongue	Significantly lower CDT, WDT, and CPT in BMS No significant differences in HPT in BMS	This pattern suggests impairments in ion channels within Aδ and C fiber nerve endings
Hartmann et al., 2017 [57]	QST: Thermal Stimuli between 5–50 °C	Left and right side of tongue	Significant higher CDT and WDT Significant lower CPT	Small fiber loss and impaired function
Kolkka et al., 2019 [59]	QST: Thermal stimuli between 10–55 °C	Lingual nerve distribution, bilateral	Higher WDT and CDT in BMS	Neuropathic pain condition due to focal SFN

Abbreviations: BMS: burning mouth syndrome; SFN: small fiber neuropathy; QST: quantitative sensory testing; WDT: warm detection threshold; CDT: cool detection threshold; HPT: heat pain threshold; CPT: cold pain threshold.

**Table 4:** Studies conducting a biopsy for SFN assessment.

Author	Test	Site	Relevant Outcomes	SFN Implication Comments
Lauria et al., 2005 [16]	Biopsy	Lateral aspect of the anterior two-thirds of the tongue	Significantly lower density of epithelial and sub-papillary nerve fibers (40%) in BMS patients compared to controls Diffuse morphological changes in epithelial and sub-papillary nerve fibers	Trigeminal small fiber sensory neuropathy The morphological changes suggest axonal degeneration.
Yilmaz et al., 2007 [17]	Biopsy	Tongue	Significantly reduced nerve fibers penetrating the epithelium in BMS patients Significantly increased TRPV1-positive fibers and NGF fibers in BMS patients Significant correlation between the baseline pain score and TRPV1 and NGF fibers, a trend toward increases of Nav1.8 fibers	increased NGF levels Up-regulation of TRPV1 and Nav1.8 nociceptor fibers suggest SFN
Beneng et al., 2010 [47]	Biopsy	Tongue (dorsal lingual mucosa, lateral to the midline in the anterior third)	Significantly increased P2X3 positive fibers in BMS patients Reduced neurofilament-staining fibers in BMS patients	P2X3 receptors are expressed predominantly in small sensory neurons, which implies that the involvement of these receptors in BMS could be part of the neuropathic pain mechanism, corroborating the presence of SFN
Beneng et al., 2010 [48]	Biopsy	Right or left dorsal lingual mucosa lateral to the midline in the anterior third of the tongue	Increased visual intensity scores for Nav1.7 in the sub-mucosal layers of the tongue, although this increase was not statistically significant between BMS and control	The increased expression of Nav1.7 could contribute to the heightened pain sensitivity and the burning sensations suggesting SFN
Penza et al., 2010 [50]	Biopsy	Anterolateral aspect of the tongue, close to the tip	Significant decrease in the density of nerve fibers in the tongue mucosa of BMS patients	This finding further strengthens the hypothesis of SFN
Borsani et al., 2014 [53]	Biopsy	Anterolateral aspect of the tongue close to the tip	Increased expression of TRPV1 throughout the full thickness of the epithelium in BMS patients	The overexpression of TRPV1 contributes to alteration of nerve fiber activity and to the burning pain characteristic of BMS. This again points towards SFN
Puhakka et al., 2016 [40]	Biopsy	Dorsal mucosa of the anterior third of the tongue	Significant decrease in intraepithelial nerve fiber density in BMS patients compared to controls Although the nerve fiber density was also lower in BMS patients compared to diabetic cadaver controls, this difference did not reach statistical significance.	This finding suggests that BMS is associated with pure peripheral small fiber damage, characteristic of SFN
Domaneschi et al., 2023 [46]	Biopsy	Tongue dorsum	Not statistically significant overexpression in Nav1.7 mRNA (3.13-fold change) Slight underexpression of Nav1.9 mRNA (0.45-fold change) Absence of detectable Nav1.8 expression	The lack of statistically significant differences in Nav1.7 expression might suggest that while this channel plays a role in the sensory processing abnormalities seen in BMS, it may not be the dominant factor in all patients Reduced expression of Nav1.9 might contribute to the altered pain perception and heightened sensitivity. This suggests that the nociceptive pathways in BMS might be dysregulated, leading to both hyposensitivity and hypersensitivity to sensory stimuli, characteristic of SFN.

Abbreviations: BMS: burning mouth syndrome; SFN: small fiber neuropathy; TRPV1: Transient Receptor Potential Vanilloid 1; NGF: Nerve Growth Factor; Nav1.7: sodium channel Nav1.7; Nav1.8: sodium channel Nav1.8; Nav1.9: sodium channel Nav1.9.

**Table 5:** Studies using BR for SFN assessment.

Author	Test	Site	Relevant Outcomes	SFN Implication Comments
Mendak et al., 2012 [52]	BR	Unilateral stimulation of lip corner	Significant differences in BR parameters in BMS patients, including prolonged latencies and irregularities. These findings are consistent with SFN, indicating mild sensory and autonomic small fiber involvement.	These findings are consistent with SFN, indicating mild sensory and autonomic small fiber involvement with concomitant central disorders.
Puhakka et al., 2016 [40]	BR	Lingual nerve distribution, bilateral	Longer BR latencies within the lingual nerve distribution in BMS patients compared to controls, although these differences did not reach statistical significance.	Some degree of small fiber involvement but highlights the variability in SFN presentation within BMS.
Kolkka et al., 2019 [59]	BR	Bilaterally lingual nerve distribution	Negative neurophysiological signs in BMS patients, even though there was no statistically significant prolongation of latencies or other typical abnormalities in BR parameters when compared to controls.	The findings are suggestive of Aδ fiber dysfunction supporting SFN. The absence of significant BR abnormalities in the majority of patients indicates that BMS might involve a more complex or varied pathophysiological process

Abbreviations: BMS: burning mouth syndrome; BR: blink reflex; SFN: small fiber neuropathy.

**Table 6:** Studies using capsaicin, artemin mRNA expression, and corneal confocal microscopy for SFN assessment.

Author	Test	Site	Relevant Outcomes	SFN Implication Comments
Just et al., 2010 [49]	Capsaicin-impregnated filter-paper strips	Dorsal anterior tongue	Higher pain and perception thresholds in BMS patients compared to controls	Peripheral trigeminal sensitivity; impaired small fiber function.
Shinoda et al., 2015 [55]	Artemin mRNA expression; scraping mucosa	Tongue	Significantly higher artemin mRNA expression in the tongue of BMS patients	Increased sensitivity of heat-responsive fibers mediated by GFRa3 signaling, which could serve as a biomarker for SFN
O’ Neill et al., 2018 [58]	CCM	Central corneal sub-basal nerve plexus	Significantly lower corneal nerve fiber density and corneal nerve fiber length in BMS patients compared with controls Significantly higher number of Langerhans cells in BMS patients compared with controls	Corneal small fiber damage suggesting a systemic involvement of SFN in BMS

Abbreviations: BMS: burning mouth syndrome; SFN: small fiber neuropathy; CCM: corneal confocal microscopy; GFRa3: Glial Cell Line-Derived Neurotrophic Factor Receptor Alpha 3. mRNA: Messenger Ribonucleic Acid.

## Data Availability

The data that supports the findings of this study are available from the corresponding author, D.A., upon reasonable request.

## Supplementary Materials

The following supporting information can be downloaded at: https://www.mdpi.com/article/10.3390/ijms252111442/s1.
