# Supplementary material for: Small Fiber Neuropathy in Burning Mouth Syndrome: A Systematic Review
Source: Int J Mol Sci. 2024 Oct 24;25(21):11442. doi: 10.3390/ijms252111442 (PMC11546372; doi:10.3390/ijms252111442)
Supplement: Supplementary file 1 [file ijms-25-11442-s001.zip › ijms-3232726-supplementary.pdf]

Database Search and Exclusion Criteria

Category	Details
Total Articles	PubMed: 17<br>Scopus: 14<br>Web of Science: 31<br>Total: 62
Duplicates Removed	16 (remaining: 46)
Removed for Other Reasons	Not topic-relevant: 13<br>Non-English: 2<br>Total: 15
Abstract Evaluation	Initial: 31<br>Excluded: 4<br>Assessed for Eligibility: 27<br>Manual Search: 8
Full-Text Assessment	Assessed: 35<br>Excluded: 15 (Conference: 3, Review: 10, No Control Group: 2)<br>Total Included: 20

Categorization for Exclusion reasons

A. Duplicates:

1. Jääskeläinen SK(1). Is burning mouth syndrome a neuropathic pain condition? Pain. 2018 Mar;159(3):610-613. doi: 10.1097/j.pain.0000000000001090.
2. Tavee J(1), Zhou L. Small fiber neuropathy: A burning problem. Cleve Clin J Med. 2009 May;76(5):297-305. doi: 10.3949/ccjm.76a.08070.

3. O'Neill F, Marshall A, Ferdousi M, Malik RA. Corneal Confocal Microscopy Detects Small-Fiber Neuropathy in Burning Mouth Syndrome: A Cross-Sectional Study. J Oral Facial Pain Headache. 2019 Summer;33(3):337–341. doi: 10.11607/ofph.2338. Epub 2019 Apr 24.

4. Puhakka A(1), Forssell H(2), Soinila S(3)(4)(5), Virtanen A(6), Röyttä M(7), Laine M(2), Tenovuo O(4)(8), Teerijoki-Oksa T(9), Jääskeläinen SK(1)(10). Oral Dis. Peripheral nervous system involvement in primary burning mouth syndrome--results of a pilot study. 2016 May;22(4):338-44. doi: 10.1111/odi.12454. Epub 2016 Feb 26.

5. Lauria G(1), Majorana A, Borgna M, Lombardi R, Penza P, Padovani A, Sapelli P. Trigeminal small-fiber sensory neuropathy causes burning mouth syndrome. Pain. 2005 Jun;115(3):332-337. doi: 10.1016/j.pain.2005.03.028. **(x3) (The same title in 3 versions with the same authors in different places)**

6. Mendak-Ziółko M(1), Konopka T, Bogucki ZA. Evaluation of select neurophysiological, clinical and psychological tests for burning mouth syndrome. Oral Surg Oral Med Oral Pathol Oral Radiol. 2012 Sep;114(3):325-32. doi: 10.1016/j.oooo.2012.04.004.

7. Birnbaum J(1). Peripheral nervous system manifestations of Sjögren syndrome: clinical patterns, diagnostic paradigms, etiopathogenesis, and therapeutic strategies. Neurologist. 2010 Sep;16(5):287-97. doi: 10.1097/NRL.0b013e3181ebe59f.

8. Kaplan I(1), Levin T, Papoiu AD, Patel N, Patel T, Calderon S, Littner M, McGlone F, Yosipovitch G. Thermal sensory and pain thresholds in the tongue and chin change with age, but are not altered in burning mouth syndrome. Skin Res Technol. 2011 May;17(2):196-200. doi: 10.1111/j.1600-0846.2010.00483.x. Epub 2011 Jan 19. **(x3)**

9. Granot M(1), Nagler RM. Association between regional idiopathic neuropathy and salivary involvement as the possible mechanism for oral sensory complaints. J Pain. 2005 Sep;6(9):581-7. doi: 10.1016/j.jpain.2005.03.010. 9 **(x3)**

10. Walega DR, Smith C, Epstein JB. Bilateral stellate ganglion blockade for recalcitrant oral pain from Burning Mouth Syndrome: a case report. J Oral Facial Pain Headache. 2014 Spring;28(2):171-5. doi: 10.11607/ofph.1165. **(x3)**

11. Jääskeläinen, Satu K. Pathophysiology of primary burning mouth syndrome (2012) Clinical Neurophysiology, 123 (1), pp. 71 - 77, Cited 203 times. DOI: 10.1016/j.clinph.2011.07.054

12. Sevilla M.R. Is Burning Mouth Syndrome Based on a Physiological Mechanism which Resembles that of Neuropathic Pain? [Comparación entre el mecanismo fisiológico del síndrome de la boca ardiente y el dolor neuropático] (2020) Odovtos - International Journal of Dental Sciences, 22 (2), pp. 15 - 18, Cited 2 times. DOI: 10.15517/IJDS.2020.39885

B.1 Removed for other reasons (Not topic-relevant)

1. Gomatos EL(1), Dulebohn SC, Rehman A(2). Sensory Neuropathy. In: StatPearls [Internet]. Treasure Island (FL): StatPearls Publishing; 2024 Jan–. 2024 Feb 25.

2. Hisama FM(1), Dib-Hajj SD(2), Waxman SG(3). SCN9A Neuropathic Pain Syndromes. In: Adam MP, Feldman J, Mirzaa GM, Pagon RA, Wallace SE, Amemiya A, editors. GeneReviews(®) [Internet]. Seattle (WA): University of Washington, Seattle; 1993–2024. 2006 May 6 [updated 2020 Jan 23].

3. Seeliger T (1), Dreyer HN(1), Siemer JM(1), Bönig L(1), Gingele S(1), Dohrn MF(2), Prenzler N(3), Ernst D(4), Witte T(4), Skripuletz T(5). Clinical and paraclinical features of small fiber neuropathy in Sjögren's syndrome. J Neurol. 2023 Feb;270(2):1004-1010. doi: 10.1007/s00415-022-11431-1. Epub 2022 Nov 4.

4. Rossi S(1)(2), Gelsomino F(3)(4), Rinaldi R(5), Muccioli L(6), Comito F(3)(4), Di Federico A(3)(4), De Giglio A(3)(4), Lamberti G(3)(4), Andrini E(3)(4), Mollica V(3)(4), D'Angelo R(5), Baccari F(5), Zenesini C(5), Madia P(5), Raschi E(7), Cortelli P(5)(6), Ardizzoni A(3)(4), Guarino M(5). Peripheral nervous system adverse events associated with immune checkpoint inhibitors. J Neurol. 2023 Jun;270(6):2975-2986. doi: 10.1007/s00415-023-11625-1. Epub 2023 Feb 17.
5. Polydefkis, M (Polydefkis, M); Griffin, JW (Griffin, JW); McArthur, J (McArthur, J)New insights into diabetic polyneuropathy. JAMA-JOURNAL OF THE AMERICAN MEDICAL ASSOCIATION Volume: 290 Issue: 10 Pages: 1371-1376 DOI: 10.1001/jama.290.10.1371

6. Navickaitė I(1), Ališauskienė M(1), Petrauskienė S(2), Žemgulytė G(1). Sarcoidosis-Associated Sensory Ganglionopathy and Harlequin Syndrome: A Case Report. Medicina (Kaunas). 2023 Aug 20;59(8):1495. doi: 10.3390/medicina59081495.

7. Birnbaum J(1). Peripheral nervous system manifestations of Sjögren syndrome: clinical patterns, diagnostic paradigms, etiopathogenesis, and therapeutic strategies. Neurologist. 2010 Sep;16(5):287-97. doi: 10.1097/NRL.0b013e3181ebe59f.

8. Vora, AR (Vora, AR); Loescher, AR (Loescher, AR); Boissonade, FM (Boissonade, FM); Robinson, PP (Robinson, PP). Ultrastructural characteristics of axons in traumatic neuromas of the human lingual nerve. JOURNAL OF OROFACIAL PAIN Volume: 19 Issue: 1 Pages: 22-33

9. Sevilla M.R. Is Burning Mouth Syndrome Based on a Physiological Mechanism which Resembles that of Neuropathic Pain? [Comparación entre el mecanismo fisiológico del síndrome de la boca ardiente y el dolor neuropático] (2020) Odovtos - International Journal of Dental Sciences, 22 (2), pp. 15 - 18, Cited 2 times. DOI: 10.15517/IJDS.2020.39885

10. Granot M(1), Nagler RM. Association between regional idiopathic neuropathy and salivary involvement as the possible mechanism for oral sensory complaints. J Pain. 2005 Sep;6(9):581-7. doi: 10.1016/j.jpain.2005.03.010. 9

11. Waldinger, MD (Waldinger, Marcel D.); de Lint, GJ (de Lint, Govert J.); Venema, PL (Venema, Pieter L.); van Gils, APG (van Gils, Ad P. G.); Schweitzer, DH (Schweitzer, Dave H.). Successful Transcutaneous Electrical Nerve Stimulation in Two Women with Restless Genital Syndrome: The Role of Aδ- and C-Nerve Fibers. JOURNAL OF SEXUAL MEDICINE Volume: 7 Issue: 3 Pages: 1190-1199 DOI: 10.1111/j.1743-6109.2009.01578.x

12. Navickaite, I (Navickaite, Ieva); Alisauskiene, M (Alisauskiene, Migle); Petrauskiene, S (Petrauskiene, Sandra); Zemgulyte, G (Zemgulyte, Gintare). Sarcoidosis-Associated Sensory Ganglionopathy and Harlequin Syndrome: A Case Report. MEDICINA-LITHUANIA Volume: 59 Issue: 8 Article Number: 1495 DOI: 10.3390/medicina59081495

13. Weimer, LH (Weimer, Louis H.); Sachdev, N (Sachdev, Noor). Update on medication-induced peripheral neuropathy. CURRENT NEUROLOGY AND NEUROSCIENCE REPORTS Volume: 9 Issue: 1

B.2 other language but English

1. Langlois V(1), Bedat Millet AL(2), Lebesnerais M(3), Miranda S(3), Marguet F(4), Benhamou Y(3), Marcorelles P(5), Lévesque H(3).Rev Med Interne. 2018 Feb;39(2):99-106. doi: 10.1016/j.revmed.2017.03.013. Epub 2017 Apr 11. Small fiber neuropathy. [Article in French]

2. Lauritano, Dott Dorina, Calzavara, D. (35755474800); Papagna, R. (15122557000); Baldoni, M. (16417009800); Bascones, A. Neuropathic syndrome in a neurophysiological and immunohistochemical study of nerve fibers in patients with burning mouth syndrome [Evidencia del síndrome neuropático en un estudio neurofisiológico e inmunohistoquímico de las fibras nerviosas en pacientes con síndrome de boca ardiente] (2003) Avances en Odontoestomatologia, 19 (2), pp. 81 - 94,

C. Excluded for specific reasons after abstract evaluation:

1. Lauritano D., Spadari F., Formaglio F., Zambellini Artini M., Salvato A. Etiopathogenic, clinical-diagnostic and therapeutic aspects of the burning mouth syndrome. Research and treatment protocols in a patient group (1998) Minerva stomatologica, 47 (6), pp. 239 - 251,

Reason for Exclusion: This study, while addressing aspects of BMS , was excluded because it did not include a control group, which is necessary for the comparative analysis required in our review

2. Azzi L., Croveri F., Pasina L., Porrini M., Vinci R., Manfredini M., Tettamanti L., Tagliabue A., Silvestre-Rangil J., Spadari F. A "burning" therapy for burning mouth syndrome: Preliminary results with the administration of topical capsaicin (2017) Journal of Biological Regulators and Homeostatic Agents, 31 (2, Supplement 1), pp. 89 - 95,

Reason for Exclusion: This study was excluded because it was focused on a preliminary investigation of capsaicin therapy for BMS, but lacked robust comparison with a control group. Preliminary studies without sufficient data on comparative outcomes do not meet the inclusion criteria for this systematic review.

3. Halac, G (Halac, Gulistan); Tekturk, P (Tekturk, Pinar); Eroglu, S (Eroglu, Saliha); Cikrikcioglu, MA (Cikrikcioglu, Mehmet Ali); Cimendur, O (Cimendur, Ozlem); Kilic, E (Kilic, Elif); Asil, T (Asil, Talip). BURNING MOUTH SYNDROME: EVALUATION OF CLINICAL AND LABORATORY FINDINGS. IDEGGYOGYASZATI SZEMLE-CLINICAL NEUROSCIENCE Volume: 69 Issue: 7-8 Pages: 269-274 DOI: 10.18071/isz.69.0269 Published Date: 2016

Reason for Exclusion: Although this study evaluated clinical and laboratory findings in BMS patients, it was excluded because it did not provide a comparison with a control group, making it insufficient for inclusion in a systematic review focused on controlled clinical studies.

4. Walega DR, Smith C, Epstein JB. Bilateral stellate ganglion blockade for recalcitrant oral pain from Burning Mouth Syndrome: a case report. J Oral Facial Pain Headache. 2014 Spring;28(2):171-5. doi: 10.11607/ofph.1165.

Reason for Exclusion: This case report was excluded because it focuses on a single intervention (stellate ganglion blockade) in an isolated case, without a broader sample size or control group for comparison. Case reports, by nature, do not meet the requirements for inclusion in a systematic review aimed at synthesizing data from larger, controlled studies.

D. Excluded after Full-Text Assessment: 15 articles [conference (3), review (10), no control group (2)]

Conference

1. Lauria, G (Lauria, G); Majorana, A (Majorana, A); Borgna, M (Borgna, M); Lombardi, R (Lombardi, R); Penza, P (Penza, P); Padovani, A (Padovani, A); Sapelli, P (Sapelli, P) Burning mouth syndrome: A new small-fiber sensory neuropathy. JOURNAL OF THE PERIPHERAL NERVOUS SYSTEM Volume: 10 Pages: 50-51 Published Date: 2005 JUL Supplement: 1

2. Laurial, G (Laurial, G.); Majorana, A (Majorana, A.); Borgnal, M (Borgnal, M.); Lombardi, R (Lombardi, R.); Penza, P (Penza, P.); Padovani, A (Padovani, A.); Sapelli, P (Sapelli, P.) Burning mouth syndrome: A new small-fiber sensory neuropathy. EUROPEAN JOURNAL OF NEUROLOGY Volume: 12 Pages: 25-25 Published Date: 2005 SEP Supplement: 2

3. Majorana, A (Majorana, A.); Lombardi, R (Lombardi, R.); Penza, P (Penza, P.); Padovani, A (Padovani, A.); Sapelli, P (Sapelli, P.); Lauria, G (Lauria, G.) Trigeminal small-fiber sensory neuropathy causes burning mouth syndrome. JOURNAL OF ORAL PATHOLOGY & MEDICINE Meeting Abstract: 251 Volume: 35 Issue: 7 Pages: 423-424 Published Date: 2006

Review

1. Russo M., Crafa P., Guglielmetti S., Franzoni L., Fiore W., Di Mario F. Burning Mouth Syndrome Etiology: A Narrative Review (2022) Journal of Gastrointestinal and Liver Diseases, 31 (2), pp. 223 - 228,

2. Jääskeläinen SK(1). Is burning mouth syndrome a neuropathic pain condition? Pain. 2018 Mar;159(3):610-613. doi: 10.1097/j.pain.0000000000001090.

3. Jääskeläinen, Satu K. Pathophysiology of primary burning mouth syndrome (2012) Clinical Neurophysiology, 123 (1), pp. 71 - 77, Cited 203 times. DOI: 10.1016/j.clinph.2011.07.054

4. Forssell, H (Forssell, H.); Jääskeläinen, S (Jaaskelainen, S.); List, T (List, T.); Svensson, P (Svensson, P.); Baad-Hansen, L (Baad-Hansen, L.). An update on pathophysiological mechanisms related to idiopathic oro-facial pain conditions with implications for management. JOURNAL OF ORAL REHABILITATION Volume: 42 Issue: 4 Pages: 300-322 DOI: 10.1111/joor.12256

5. Tavee J(1), Zhou L. Small fiber neuropathy: A burning problem. Cleve Clin J Med. 2009 May;76(5):297-305. doi: 10.3949/ccjm.76a.08070.

6. Jääskeläinen, SK (Jaaskelainen, Satu K.); Woda, A (Woda, Alain). Burning mouth syndrome. CEPHALALGIA Volume: 37 Issue: 7 Pages: 627-647 DOI: 10.1177/0333102417694883 Published Date: 2017

7. Orliaguet, M (Orliaguet, Marie); Misery, L (Misery, Laurent). Neuropathic and Psychogenic Components of Burning Mouth Syndrome: A Systematic Review. BIOMOLECULES Volume: 11 Issue: 8 Article Number: 1237 DOI: 10.3390/biom11081237 Published Date: 2021

8. Klasser, GD (Klasser, Gary D.); Grushka, M (Grushka, Miriam); Su, N (Su, Nan). Burning Mouth Syndrome. ORAL AND MAXILLOFACIAL SURGERY CLINICS OF NORTH AMERICA Volume: 28 Issue: 3 Pages: 381-396 DOI: 10.1016/j.coms.2016.03.005 Published Date: 2016

9. Prakash, S (Prakash, Sanjay); Ahuja, S (Ahuja, Sunil); Rathod, C (Rathod, Chirag). Dopa responsive burning mouth syndrome: Restless mouth syndrome or oral variant of restless legs syndrome? JOURNAL OF THE NEUROLOGICAL SCIENCES Volume: 320 Issue: 1-2 Pages: 156 160 DOI: 10.1016/j.jns.2012.07.007 Published Date: 2012

10. Carreño-Hernández, I (Carreno-Hernandez, Isabel); Cassol-Spanemberg, J (Cassol-Spanemberg, Juliana); de Rivera-Campillo, ER (de Rivera-Campillo, Eugenia Rodriguez); Estrugo-Devesa, A (Estrugo Devesa, Albert); López-López, J (Lopez-Lopez, Jose). Is Burning Mouth Syndrome a Neuropathic Pain Disorder? A Systematic Review. JOURNAL OF ORAL & FACIAL PAIN AND HEADACHE Volume: 35 Issue: 3 Pages: 218 229 DOI: 10.11607/ofph.2861 Published Date: 2021

No control group

1. Forssell H(1), Jääskeläinen S, Tenovuo O, Hinkka S. Sensory dysfunction in burning mouth syndrome. Pain. 2002 Sep;99(1-2):41-7. doi: 10.1016/s0304-3959(02)00052-0.

2. Treldal C, Petersen J, Mogensen S, Therkildsen C, Jacobsen J, Andersen O, Pedersen AML. Characterization of burning mouth syndrome profiles based on response to a local anaesthetic lozenge. Oral Dis. 2020 Apr;26(3):656-669. doi: 10.1111/odi.13267. (there was a control group, but there was no data published regarding the comparison with the control group)
